# Evaluation of the Effectiveness of Tai Chi versus Brisk Walking in Reducing Cardiovascular Risk Factors: Protocol for a Randomized Controlled Trial

**DOI:** 10.3390/ijerph13070682

**Published:** 2016-07-05

**Authors:** Aileen W. K. Chan, Janet W. H. Sit, Sek Ying Chair, Doris Y. P. Leung, Diana T. F. Lee, Eliza M. L. Wong, Lawrence C. W. Fung

**Affiliations:** 1 The Nethersole School of Nursing, The Chinese University of Hong Kong, New Territories, Hong Kong, China; janet.sit@cuhk.edu.hk (J.W.H.S.); sychair@cuhk.edu.hk (S.Y.C.); dorisleung@cuhk.edu.hk (D.Y.P.L.); tzefanlee@cuhk.edu.hk (D.T.F.L.); elizawong@cuhk.edu.hk (E.M.L.W.); 2 Physiotherapy Department, Kwong Wah Hospital, 25 Waterloo Road, Yaumatei, Kowloon, Hong Kong, China; lcwfung@ha.org.hk

**Keywords:** brisk walking, cardiovascular risk factors, Tai Chi

## Abstract

Physical inactivity is one of the major modifiable lifestyle risk factors for cardiovascular disease (CVD). This protocol aims to evaluate the effectiveness of Tai Chi versus brisk walking in reducing CVD risk factors. This is a randomized controlled trial with three arms, namely, Tai Chi group, walking group, and control group. The Tai Chi group will receive Tai Chi training, which consists of two 60-min sessions each week for three months, and self-practice for 30 min every day. The walking group will perform brisk walking for 30 min every day. The control group will receive their usual care. 246 subjects with CVD risk factors will be recruited from two outpatient clinics. The primary outcome is blood pressure. Secondary outcomes include fasting blood for lipid profile, sugar and glycated haemoglobin (HbA1c); body mass index, waist circumference, body fat percentage; perceived stress level and quality of life. Data collections will be conducted at baseline, 3-month, 6-month and 9-month. Generalized estimating equations model will be used to compare the changes in outcomes across time between groups. It is expected that both the Tai Chi and walking groups could maintain better health and have improved quality of life, and that Tai Chi will be more effective than brisk walking in reducing CVD risk factors.

## 1. Introduction

According to the World Health Organization [1], an estimated 17.3 million people died from cardiovascular diseases (CVD) in 2008. By 2030, more than 23 million people will die annually from CVD. In Hong Kong, CVD is the fourth leading cause of death, and more than 3000 people die from CVD each year [2]. The prevalence of CVD risk factors is rapidly increasing in developing and developed countries. Individuals with CVD risk factors have a twofold chance of death, threefold chance of developing CVD, and fivefold chance of suffering from type 2 diabetes mellitus [3]. This development imposes a significant health burden on society. Therefore, CVD is clearly an important and common public health problem, and preventive measures need to be implemented to minimize the risk factors contributing to CVD.

The core CVD risk factors include hypertension, hyperglycemia, dyslipidemia, and obesity [4]. Psychosocial stressors affect the quality of life and associate with increased CVD risk factors [5]. Physical inactivity is one of the major lifestyle risk factors for CVD [1]. Staying physically active is always recommended because regular physical exercise can increase insulin sensitivity, improve lipid levels, lower blood pressure (BP), reduce weight, and lower the risk of CVD [3].

In Hong Kong, the Behavioural Risk Factor Survey conducted by the Department of Health in April 2010 found that the level of physical activity of the residents was clearly not enough for optimal health gain [6]. Studies have shown that interventions to promote regular physical activity are cost-effective in the prevention and the control of CVD risk factors [7]. For a home program, walking is usually adopted for exercise training. Walking has the potential to play a key role in the prevention of CVD [8]. Studies have indicated that the physical benefits of walking are related to consistent adherence and regular exercise. However, adherence to regular walking is still inadequate [7,9]. Among the different types of exercise practiced in the community, Tai Chi can prevent and control CVD risk factors because of its advantages for promoting health. Moreover, participants are relatively more compliant to regular Tai Chi exercise [10].

Tai Chi is a form of martial arts that originated in China in the 16th century. Tai Chi is a therapeutic exercise based on Chinese medicine, and the practice involves the recognition, development, and use of *Qi*. According to the traditional Chinese medicine (TCM) theory, *Qi* is the fundamental energy that sustains life. One purpose of Tai Chi is to balance Yin and Yang and to promote good circulation of *Qi* in the body. A variety of cardiopulmonary fitness indicators has been examined for Tai Chi. Research has demonstrated that Tai Chi has beneficial effects on patients with heart disease [11], metabolic control in type 2 diabetes [12], and on the reduction of BP [13], and emotional stress [14]. Cardiopulmonary benefits of Tai Chi may be partially explained as a response to aerobic exercise. Studies have shown that Tai Chi can be classified as moderate exercise because its intensity does not exceed 55% of maximal oxygen intake [15].

Tai Chi has a long history and is popular among the Chinese population. Therefore, it may be perceived as more culturally appropriate for the Chinese people than other forms of conventional exercise. Tai Chi is a self-learned skill and is easily accessible in the community. Once Tai Chi is learned, people can perform it conveniently at any time and at any place without the need for supervision. From a social perspective, given the ever-rising costs in the healthcare system, adopting a low-cost behavioral modality to promote health and to prevent illnesses is particularly attractive.

Today, health benefits from practicing Tai Chi have gained attention. The focus is not only on how it can alleviate the signs and symptoms of chronic diseases and psychological illnesses, but also on how it can prevent the development of chronic diseases and promote health. Although there are literatures showing that Tai Chi has beneficial effects on people with cardiovascular risk factors [16,17,18], a recent review by Hartley et al. [19] identified 13 trials using Tai Chi for primary prevention of CVD. Authors pointed out that there were some suggestions of beneficial effects of Tai Chi on CVD risk factors but this was not consistent across all studies. Most trials were relatively small (*n* ≤ 88) and short term (≤6 months). They concluded that the effectiveness of Tai Chi for the primary prevention of CVD risk factors was inconclusive [19].

For a home program, walking is usually adopted for exercise training. However, adherence to regular brisk walking is still inadequate [7]. Hence, current literature lacks evidence to support the sustaining beneficial effects of brisk walking on CVD risk reduction. Tai Chi is a safe exercise that requires an energy expenditure that is similar to brisk walking [20]. However, Tai Chi is not known to provide the same cardiovascular training effect as brisk walking. Comparing the health benefits between Tai Chi and brisk walking, particularly to people with CVD risk factors, have been rarely studied in healthcare research. To address the gaps of knowledge, this study aims to compare the effects of Tai Chi with brisk walking in reducing modifiable CVD risk factors. If Tai Chi is as good as or even better than regular brisk walking, this would be of considerable public health importance as it is socially acceptable and attractive and might well be better taken up and sustained than brisk walking.

## 2. Materials and Methods

### 2.1. Subjects

Potential subjects will be recruited from two outpatient clinics. Informed consent will be obtained from every eligible patient who agrees to participate.

Inclusion criteria: Subjects who:

(1) Have systolic blood pressure (SBP) > 140 mmHg or diastolic blood pressure (DBP) > 90 mmHg or are taking blood pressure medication, and
(2) Meet at least two but not more than three of the following CVD risk factors that are defined according to the American Heart Association [4]:
  (a) Males older than 45 years
  (b) Females older than 55 years or postmenopausal
  (c) Smoker
  (d) Fasting cholesterol level > 6.2 mmol/L
  (e) Have diabetes or taking medicine to control blood sugar
  (f) Physically inactive (get <30 min of physical activity for at least 3 days a week)
  (g) Body mass index (BMI) >25 kg/m^2^ or waist circumference ≥90 cm (men) or ≥80 cm (women)

The reason for limiting the CVD risk factors to two but not more than three is to minimize the heterogeneity of subjects. If subjects are included with any CVD risk factors, this will produce some heterogeneity in the sample, with some having more risk factors than others, which may impact the results.

Exclusion criteria: Subjects who:

(1) Have developed cardiovascular disease
(2) Suffered from severe sensory or cognitive impairment
(3) Cannot walk independently
(4) Have musculoskeletal disorders or other disabling diseases that may limit the practice of Tai Chi
(5) Have previous training and practiced Tai Chi within six months prior to the commencement of the study

### 2.2. Study Design

This study will employ randomized controlled trial. Potential subjects will be randomized to enter one of the three groups, namely, Tai Chi, walking, and control. A pilot study has been conducted to test the feasibility of the recruitment process, data collection process, study instruments and intervention process. Interrater reliability test among research assistants was also performed to ensure consistency of the data collection process. Twenty-five participants were recruited for the pilot study. The recruitment process lasted for five weeks (20 participants per month) and it went smoothly. Participant responses toward the data collection instruments generally indicated that the questionnaires were understandable and that the physiological tests were easy to perform.

#### Sample Size and Randomization

A previous study conducted in the Asia-Pacific region reported that SBP is an important marker of risk of CVD in people with and without diabetes. A given reduction in SBP is likely to have similar relative effect on reducing the risk of a cardiovascular event, regardless of diabetes status [21]. SBP is therefore being set as the primary outcome and for sample size calculation.

A previous local study on a Tai Chi program reported a mean reduction of 15.6 mmHg (standard deviation, SD = 7.9) in the intervention group and a mean increase of 6.4 mmHg (SD = 10.5) in the control group [22]. Another pre-post study showed a mean reduction of 8.7 mmHg (SD = 15.6) in SBP after receiving a walking program in obese postmenopausal women [23]. We based our sample size calculation conservatively on discriminating among a mean SBP reduction of 15.5 mmHg in the Tai Chi group, 4.0 mmHg in the control group, and 9.75 mmHg (the grand mean) in the walking group. Further conservatively assuming an SD of 20.0, G*Power gives that 61 subjects per arm will be needed to achieve a power of 80% at a significance level of 5%. To further take into account a potential attrition rate of 25% [12], 246 subjects (82/arm) will be targeted.

A computer-based randomizer will be used for random allocation of subjects [24]. Eligible subjects will be randomly assigned to the three groups in 1:1:1 ratio according to their sequence of enrolment into the study and the corresponding sequentially listed group identifiers generated by the randomizer in advance. The random group identifiers list will be stored in a password-protected computer and only be accessible by the staff who are responsible for group allocation.

### 2.3. Intervention Protocol

#### 2.3.1. Tai Chi Group

Participants in the intervention group will attend a 60-min Tai Chi practice session twice a week for three months. This duration is supported by previous studies that even a short duration of 12-week intervention of Tai Chi can improve physical performance of healthy elderly people [25] and patients with chronic illnesses [10]. We choose three months for our subjects as the training period to balance the time for optimal result and the issue of compliance. Based on experience in a chronic illness rehabilitation program, intensive intervention beyond 12 weeks would cause participants to drop out of the study. High attrition rate would seriously affect the validity of the study. Moreover, normally the Tai Chi instructor will teach a new form in each session after revising the taught forms. It needs 24 sessions to complete the 24 forms, which accounts for three months.

The Tai Chi program is a 24-form Yang style Tai Chi, which was modified and tailor-made for our target population by two Tai Chi experts. This modified 24-form Tai Chi is designed for easy learning and mastery in a shorter period. The modified forms were also reviewed by an experienced physiotherapist to confirm their safety and feasibility to be used on the target participants. During the Tai Chi practice sessions, an experienced Tai Chi instructor will lead the participants who will replicate the motions, postures, and speed of movement. Participants will be encouraged to perform self-practice at home for 30 min per day, for at least 5 days per week. A self-recorded logbook will be used to monitor participants' adherence. Participants will be required to record their frequency and duration of self-practice in a logbook on a daily basis. The logbook will be checked by the research assistant weekly at the Tai Chi class. A compliance rate of ≥80% is considered as adherence.

#### 2.3.2. Walking Group

As recommended by the Centre for Health Protection, aerobic exercise should be done at least at moderate intensity, such as brisk walking, for at least five days per week [26]. Moderate intensity activity could be expressed as 50% to 70% of maximum heart rate. Maximum heart rate is estimated by “220 minus age” [27]. Participants in the walking group will be recommended to perform brisk walking (5 to 6 km/h) for 30 min every day, for at least 5 days per week. They may practice continuously or intermittently in bouts of at least 10 min to accumulate 30 min [27]. Participants will be given a pulse watch to measure their heart rate during brisk walking. They will be advised to achieve an individualized heart rate equivalent to moderate intensity exercise according to their age. A diary will also be given to the participants to record their heart rate, frequency and duration of brisk walking. The research assistant will contact participants by phone weekly to monitor their adherence. A compliance rate of ≥80% is considered as adherence.

#### 2.3.3. Control Group

Participants in the control group will be advised to maintain their routine activities. No extra exercise will be recommended. During the study period, all subjects will continue their prescribed medical treatments, if any. To enhance the internal validity of the study findings, participants in the walking and control groups will be arranged to join non-health-related social activities regularly for three months. The purpose of maintaining regular gatherings for the participants is to balance the psychosocial effect of weekly gatherings of the Tai Chi group during the process of Tai Chi training.

### 2.4. Outcome Measurements

#### 2.4.1. Primary Outcomes

(a) BP: Two measurements of BP will be measured from the seated position after the participant has rested for 10 min. The average of the two values will be used for study purposes. SBP and DBP will be recorded. Only one digital blood pressure monitor will be used for all BP measurements for all participants. Coefficients of variation (CV) of SBP and DBP were reported <10% in a previous study [22]. CV is a unit-less measure that depicts the size of the SD relative to its mean.

#### 2.4.2. Secondary Outcomes

(a) After an 8- to 10-h fasting, blood samples will be taken by using a finger-stick for the following measurements using auto-analyzers:
  (i) Fasting blood sugar (FBS)
  (ii) Glycated hemoglobin (HbA1c)
  (iii) Total cholesterol (TC), triglyceride (TG), high-density lipoprotein (HDL) and low-density lipoprotein (LDL). CVs of all these six variables were <0.31 in previous studies [22,28]
(b) BMI will be calculated as weight (kg) divided by the square of height (m). Height and weight (measured to the nearest 0.1 kg) will be measured with the subject wearing light clothing without shoes
(c) Waist circumference will be measured to within 1 mm by using a plastic measuring tape at midway between the lowest rib and the iliac crest with the subject standing at the end of gentle expiration. CV of waist circumference was reported <0.13 in a previous study [29].
(d) Frequency and duration of self-practice of Tai Chi/brisk walking from a self-recorded logbook
(e) Perceived stress scale (PSS-10)

Ten self-report items measure the degree to which situations in one’s life are appraised as stressful and the current levels of experienced stress in the last month. Summative scores range from 0 to 40, with a higher score indicating higher stress level. These scores have been used as outcome measures of experienced levels of stress. The psychometric properties of the Chinese version of PSS-10 were satisfactory with Cronbach’s alphas >0.75 in a local study for Chinese cardiac patients [30]. CVs of PSS-10 were reported to be 0.44 at baseline, 0.50 at 6 weeks and 0.48 at 12 weeks in a previous study [31].

(f) Quality of life: Short-form 12 health survey (SF-12v2)

The SF-12v2 is a popular generic health-related quality of life measure. This 12-item questionnaire measures functional health and well-being from the perspective of clients. The survey has favorable internal consistency and test-retest reliabilities (from 0.67 to 0.82). SF-12v2 is valid, reliable, and sensitive for the Chinese population with CVs for both physical and mental component summary <0.2 [32].

### 2.5. Data Collection Procedure

To minimize researcher bias, research assistants (RA) who are responsible for data collection will be blinded to the group allocation of the subjects. They will receive a briefing session explaining procedures for data collection. The RAs will also be trained in using the finger-stick for collecting blood samples, and in the procedures of using the auto-analyzers. Inter-rater reliability of the data collection procedures among the RAs will be examined.

**Figure 1:**
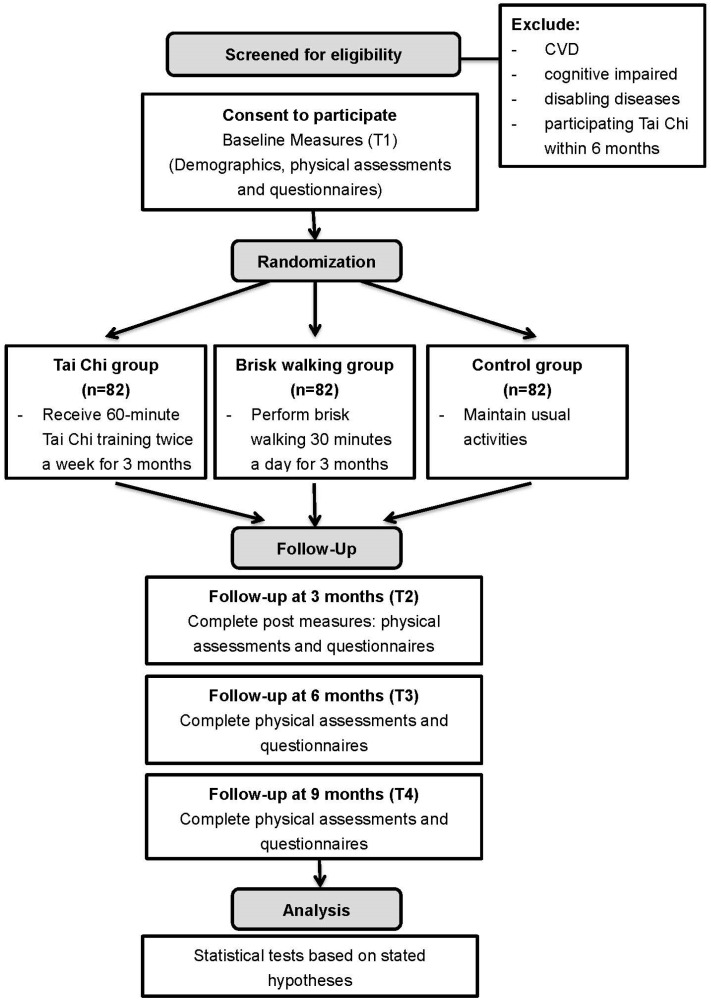
Design of the study.

Informed consent will be obtained from the eligible subjects. Data will be collected through physical assessments and questionnaires. The medical records of all participants will be reviewed for demographic information, medical history, and comorbidities. Pre-test at baseline (T1) and post-test at three months (T2) measurements will be obtained. To monitor the long-term compliance behaviors of subjects on Tai Chi practice and walking exercise, follow-up evaluations will be conducted at six months (T3) and nine months (T4). Twenty participants per month are anticipated to participate in the main study. Therefore, to recruit 246 participants, 12 months will be required to recruit the required number of participants, and another 9 months are needed to complete all T4 data collection points. Figure 1 shows the flowchart of the study design in accordance with the CONSORT guidelines for randomized controlled trial (RCT) [33].

### 2.6. Data Analysis

(1) For demographic data, frequency and percentage will be used to summarize and present categorical variables, whereas mean and SD will be used for continuous variables. Normality of continuous variables will be examined using skewness and kurtosis statistics and normal probability plot. The baseline characteristics among the three groups will be compared using chi-square test and one-way analysis of variance (ANOVA).
(2) Generalized estimating equations (GEE) models with appropriate link function and distribution assumption, will be used to compare the changes in outcome variables across time among the three groups with adjustment for those baseline characteristics showing statistically significant differences among the groups. In the GEE model for each outcome, two dummy variables will be set to correspond the three groups with the control as reference. Another three dummy variables will be set to represent the four time points (T1 to T4) with the baseline (T1) as the reference. The interaction-terms of group by time will also be included in the GEE models to assess the differential changes of the outcomes across time and between group. All primary and secondary outcomes will be compared between the three groups on the basis of the intention-to-treat (ITT) principle. The GEE model can account for intra-correlated repeated measures data and accommodate missing data caused by dropouts, provided the data is missing at random [34], and thus is particularly suitable for ITT analysis, without the need for imputation of missing data.

All data analyses will be performed using the latest version of IBM SPSS (IBM Corp., Armonk, NY, USA). Level of statistical significance will be set at 0.05.

### 2.7. Ethical Considerations

The ethical consideration principles will adhere to the Declaration of Helsinki and International Conference on Harmonisation of Technical Requirements for Registration of Pharmaceuticals for Human Use—good clinical practice (ICH-GCP). Ethics approval has been obtained from the Joint Clinical Research Ethics Committee of the Chinese University of Hong Kong and New Territory East Cluster and the Kowloon West Cluster. The ethical code for the RCT is CRE = 2014.131-T. Clients are voluntary participants in the study, and they will be informed about the purpose of the study. A written informed consent, which will include the research title, purpose, explanation of the research, and the procedures of the study, will be obtained from each eligible participant. Risks and benefits are also explained clearly to the participants. Side effects arising from Tai Chi and walking are rare. Normal reactions, including dyspnea and tiredness, that may occur are easily resolved by resting. Each participant will be given the opportunity to ask questions and he or she is free to refuse to answer any questions and any assessments, and may withdraw from the study at any time. Participants will be protected from discomfort and harm during the study. Furthermore, anonymity and confidentiality of the participants will be observed.

### 2.8. Dissemination

The findings of this study will be reported to the Health and Medical Research Fund Board, Food and Health Bureau, Hong Kong Special Administrative Region which is the funding body of this trial. The findings will be presented within hospitals and published as internal medical newsletter. The findings will also be reported to the Hospital Authority which is in charge of hospital policies. Therefore, health professionals will be informed of the findings and consider to develop strategies in order to provide care to meet the patients’ need. In addition, the findings will be presented in international conferences and the research manuscript will be published in international peer-reviewed journals. Furthermore, as the study involves individual’s participation, the results will also be disseminated to the general public.

## 3. Discussion

CVD is one of the major health problems worldwide. It impacts on not only the individual’s health but also the economic burden of the health care system. Physical activity and exercise are the cornerstone in reducing the CVD risk factors. Among the different types of exercise practiced in the community, walking is one of the most popular aerobic activities. Walking is an appropriate exercise for people of all ages. Studies have indicated that physical benefits of walking are related to consistent adherence and regular exercise [9]. However, adherence to regular walking exercise is still inadequate [7].

With the favorable results obtained from previous research studies [28,31], Tai Chi appears to be a possible intervention to reduce CVD risk factors. Tai Chi means “supreme ultimate” in Chinese [35]. It represents a reference to the philosophical bipolar concept of Yin and Yang that underlies traditional Chinese medicine theory [36]. Tai Chi was originally developed both as a martial art and as a form of meditation. The practice of Tai Chi as meditative movement is expected to elicit internal functional balance for healing, stress neutralization, longevity, and personal harmony [37]. Tai Chi is a well-known form of exercise for refining *Qi* and for enhancing physiological and psychological functions.

Studies have shown that Tai Chi can be classified as moderate exercise because its intensity does not exceed 55% of maximal oxygen intake [15]; thus, it is most suitable and beneficial to cardiopulmonary promotion. Klein and Adams [38] revealed that the slower pace and absence of any explosive or high-impact movements in the Tai Chi is primarily beneficial for health. It could improve energy through slow, gentle body movement, controlled rhythmic breathing, relaxation, and mindful awareness.

A variety of cardiopulmonary fitness indicators has been examined for Tai Chi. A review of literature by Taylor-Piliae [39] showed that simplified forms of Tai Chi are ideal for people with impaired health conditions, including those with heart disease and the elderly. Consistent findings were observed in the significant reduction in blood pressure reported in multiple studies, especially when Tai Chi [13,22] was compared to inactive control groups. Other Tai Chi studies, however, have utilized active control interventions with low to moderate levels of physical activity, which showed positive changes for both groups, but without significant differences between groups [40]. This showed preliminary evidence that Tai Chi achieved similar results to conventional exercise. Studies that failed to demonstrate significant improvements following Tai Chi [41,42,43] had fewer participants, and distinguishing whether nonsignificant findings in cardiopulmonary fitness were due to chronic and weakening illnesses or if they were a result of the limited statistical power with small sample size proved difficult. Cardiopulmonary benefits of Tai Chi may partially be explained as response to aerobic exercise. Understanding the benefits of Tai Chi in reducing CVD risk factors is of great value in guiding the prescription of an effective intervention to address the health needs of this population. Tai Chi’s easy application in a clinical setting has particular relevance to the current situation because healthcare resources, in terms of both manpower and funding, are tight. With a mounting demand for care of people with CVD, this study is timely; its findings should provide important implications for advancing the quality of care delivered by health professionals.

### Strengths and Limitations of This Study

This trial tends to establish the purported cause-and-effect relationship between the study interventions and the outcomes. Its strengths are attributed to its randomness in the involvement of independent control and experimental groups. The study design allows a rigorous control of the internal validity of the study. The consistency of the Tai Chi program in terms of content, intensity, and physical environment is maintained throughout the intervention period. The consistency of the Tai Chi training sessions is also maintained by having the same instructor to conduct all the sessions to minimize variations among different instructors. To balance the psychosocial effects of weekly gatherings of the Tai Chi training in the Tai Chi group, regular social activities will be arranged for the brisk walking group and the control group. This will strengthen the internal validity of the study by neutralizing confounding effects of the weekly gathering in the Tai Chi group. However, the study is limited by the inability to adopt a double-blind method. Blinding the participants from the information about their group status is not possible. To address the limitation, a single-blind method was adopted in which the RAs for data collection will be blinded to the participants’ group allocation.

## 4. Conclusions

CVD is a global issue in both developing and developed countries. People with CVD risk factors are associated with increased morbidity and mortality. With the favorable results obtained from previous research studies, Tai Chi appears to be a possible intervention in reducing CVD risk factors. The applicability of Tai Chi among people with CVD risk factors deserves special attention. Promotion of physiological and psychosocial health among the people with CVD risk factors represents an important goal of patient care, and evaluating the beneficial effect and clinical applicability of Tai Chi in this particular population is worthwhile. The overall aim of the study seeks to establish evidence on comparing the effectiveness and sustainability of Tai Chi and brisk walking in reducing CVD risk factors. If Tai Chi is as good as or even better than brisk walking, this would be of considerable public health importance.
